# PAIN THAT LIMITS PHYSICAL ACTIVITY AFFECTS COGNITION IN ADULTS

**DOI:** 10.1093/geroni/igz038.2627

**Published:** 2019-11-08

**Authors:** Amanda F Elliott, Ann Horgas

**Affiliations:** University of Florida, Gainesville, Florida, United States

## Abstract

Reduced cognitive skills have been observed in adults with chronic pain. The purpose of this study was to explore the relationship between pain and cognition in adults utilizing a large national sample. This was a cross-sectional study employing the 2015 wave of the Behavioral Risk Factor Surveillance System (BRFSS). A total of 134,058 adults had complete data on the variables of interest for this study. Half of the sample (50%; n = 66,479) reported having joint symptoms or arthritis that was physically limiting and 16% (n = 21,976) reported having difficulty concentrating, remembering, or making decisions. Twelve percent (n = 16,537) of this sample reported having both physically limiting pain and difficulty concentrating and remembering. Chi-square analyses reveal a statistically significant association between pain and cognition in this sample [X2(1, N= 134,058) = 6925.5, p<.01], with a small to medium effect size (phi=0.227). This study provides support that pain is associated with difficulty concentrating and remembering in adults. Pain is a common persistent symptom among older adults and its effect on cognitive functioning should be noted. Effective pain treatment strategies are warranted to help reduce the cognitive burden of chronic pain. In turn, in older adults who are experiencing concentration or memory problems, health care providers should assess the individual’s pain as a possible contributor. Additional studies assessing both pain and cognition are warranted, especially looking at the relationship of these conditions over time.

